# Assessment of Chemotherapy-Induced Cardiac Dysfunction in Breast Cancer Patients: A Prospective Study

**DOI:** 10.7759/cureus.59461

**Published:** 2024-05-01

**Authors:** Jasvinder Singh, Syed Abid Iqbal, Sahini Gajula, Prithvi Raghavan, Shreyaa Rajpal, Aadil Khan

**Affiliations:** 1 Cardiology and Electrophysiology, Asian Institute of Gastroenterology (AIG) Hospital, Hyderabad, IND; 2 Internal Medicine, Asian Institute of Gastroenterology (AIG) Hospital, Hyderabad, IND; 3 Internal Medicine, Gandhi Medical College and Hospital, Secunderabad, IND; 4 Internal Medicine, Osmania Medical College, Hyderabad, IND; 5 Trauma Surgery, OSF St Francis Medical Centre, University of Illinois College of Medicine, Peoria, USA; 6 Cardiology, University of Illinois Chicago, Chicago, USA; 7 Internal Medicine, Lala Lajpat Rai (LLR) Hospital, Kanpur, IND

**Keywords:** heart failure (hf), global longitudinal strain (gls), acute cardio toxicity, left ventricular dysfunction (lvd), chemotherapy-related cardiac dysfunction (ctrcd)

## Abstract

Background

Advances in cancer treatment have markedly improved survival rates but have also heightened morbidity due to treatment-related side effects. Despite this, the literature remains scarce on predicting the incidence of acute cardiac toxicity resulting from chemotherapy. We conducted a prospective evaluation to assess the incidence, timing, clinical correlates, global longitudinal strain (GLS), and response to heart failure (HF) therapy in patients experiencing cardiotoxicity.

Aims and objectives

Our study aimed to assess the cardiovascular complications of cancer therapy in breast cancer patients, with particular emphasis on therapy-related cardiac dysfunction.

Materials and methods

We conducted a prospective observational study to detect chemotherapy-related cardiac dysfunction (CTRCD) in breast cancer patients attending the outpatient department (OPD) or admitted to Dayanand Medical College and Hospital (DMCH), Ludhiana, Punjab, between March 1, 2020, and October 31, 2021. We assessed left ventricular ejection fraction (LVEF) at baseline, mid-chemotherapy, and post-chemotherapy. Patients who developed left ventricular dysfunction (LVD) had their chemotherapy regimen modified and were initiated on HF therapy.

Results

Ninety-seven patients (mean age: 50.74±10.30 years) were enrolled and categorized into the LVD group (n=13) and non-LVD group (n=84). CTRCD developed in 13 patients (13.4%). Patients with estrogen receptor (ER) positive, progesterone receptor (PR) positive, and human epidermal growth factor receptor 2 (HER2) positive status, as well as those in cancer stages III and IV, are at higher risk of developing LV dysfunction. Among the 13 patients, 10 (77%) experienced complete recovery, while three (23%) had partial recovery. Markers for partial recovery included cancer stages III-IV, younger age, lower body mass index (BMI), lower radiotherapy dosage, lower mean chemotherapy dosage, and left breast involvement.

Conclusion

Our findings suggest that acute cardiotoxicity is not linked to the cumulative dose of anthracyclines. Early detection, modification of chemotherapy regimens, and prompt initiation of CTRCD therapy can lead to substantial recovery of cardiac dysfunction.

## Introduction

Undoubtedly, advancements in cancer diagnosis and treatment have significantly improved cancer patients' survival rates. The fact that cancers are increasing at an alarming rate is no exaggeration. According to the US National Cancer Institute, estimates suggest that 13.7 million cancer survivors were alive in 2012, and this number will approach 18 million by 2022 [1]. Breast cancer is the leading cause of cancer death in women (15.0%), followed by lung cancer (13.8%) and colorectal cancer (9.5%), with cervical cancer ranking fourth (7.5%) [2]. However, cancer therapy-related adverse effects are a matter of concern among physicians involved in the care of cancer patients. Some cancer treatments have adverse side effects, of which cardiovascular complications are the most important and worrisome. Thus, chemotherapy is now proven to be a "double-edged sword." Cancer patients who were successfully treated with chemotherapy are suffering from cardiovascular diseases. For instance, anthracyclines, trastuzumab, and some tyrosine kinase inhibitors have a detrimental effect on myocardial function.

In India, according to GLOBOCAN 2018, overall breast cancer is the most observed cancer (14% of total cases) and is a leading cause of death (11.1%) [3]. Among females, it is also the most diagnosed cancer (27.7%) and a leading cause of death (23.5%). The age-adjusted number of incident cases in India is estimated to rise from 1.15 million to 1.9 million, and the number of deaths is estimated to rise from 0.78 million to 1.33 million by 2040 [4]. Breast cancer is currently a major public health and economic issue because the incidence of breast cancer is increasing. Advancements in chemotherapy and multidisciplinary approaches have made breast cancer curable [5]. Mortality rates have dropped significantly. Ensuring the safety of oncologic therapies is paramount with increasing life expectancy.

Anthracyclines (e.g., doxorubicin, c) and taxanes (e.g., paclitaxel) stand as major treatments for breast cancer, with anthracyclines notably effective. Anthracycline-induced cardiotoxicity, divided into acute, early-onset, and late-onset, poses risks to breast cancer patients. Acute toxicity, occurring within 14 days post-treatment, often manifests as supraventricular arrhythmia and transient left ventricular dysfunction, affecting <1% of patients and typically reversible. Early-onset cardiotoxicity emerges within a year, while late-onset can surface after about seven years. Cardiotoxicity types include irreversible type 1, associated with anthracyclines' direct myocardial damage due to cumulative doses, and reversible type 2, commonly linked to agents like trastuzumab. Doxorubicin and epirubicin, extensively used, underscore the risk of cardiotoxicity, severely impacting patient quality of life. Recommended cumulative doses for doxorubicin and epirubicin are 400 mg/m2 and 900 mg/m2, respectively, with a 5% congestive heart failure occurrence at 400 mg/m2 for doxorubicin. The oxidative stress hypothesis elucidates anthracycline-induced toxicity, emphasizing reactive oxygen species generation and lipid peroxidation damaging cardiomyocyte membranes [6-9]. In a study by Cardinal et al. involving 2625 patients (mean follow-up: 5.2 years), cardiotoxicity incidence after anthracycline treatment was 9%, with 98% occurring within the first asymptomatic year [7,10]. Risk factors include cumulative dose, infusion regimen, preexisting cardiac conditions, hypertension, concomitant chemotherapy, and age over 65 years [11]. Cyclophosphamide, cisplatin, ifosfamide, and taxanes (paclitaxel and docetaxel) are among the conventional chemotherapies capable of inducing myocardial dysfunction and heart failure. In combination with or after anthracycline, it also appears to increase the incidence of heart failure; however, the contribution of individual drugs in multidrug regimens is difficult to assess.

Immunotherapies and targeted therapies have enhanced the effectiveness of cancer treatments. Blocking human epidermal growth factor receptor 2 (HER 3) signaling using antibodies (such as trastuzumab, pertuzumab, and trastuzumab-emtasine) has improved outcomes for patients with HER2-positive breast cancer when combined with chemotherapy. Large-scale clinical trials indicate cardiac dysfunction rates ranging from 7% to 34%, with heart failure rates between 0% and 4%. When trastuzumab is administered alongside anthracyclines, heart failure rates are 6.2% after one year and <20.1% after five years. Among patients receiving trastuzumab, cardiotoxicity is the primary cause of treatment interruption in 13.5% of cases [9,12].

Radiation exposure is associated with an increased risk of ischemic heart disease in patients with breast cancer. Radiation to the left breast is of particular concern given its anatomical proximity to the heart, and studies have shown increased cardiovascular events in patients receiving left-sided radiotherapy compared to the right side. Marked interstitial myocardial fibrosis is common in radiation-induced cardiotoxicity [9].

Preventive strategies for cardiotoxicity involve early diagnosis, limiting cumulative doses, prolonged administration time (infusion over 24 - 96 hours), strengthening the monitoring of cardiotoxicity, and the use of liposomal anthracycline. Non-pharmacological measures should be promoted, including a healthy diet, smoking cessation, regular exercise, and weight control. Prior to starting potential cardiotoxic chemotherapy, risk stratification and echocardiography should be done to determine the baseline left ventricular function. Early detection of decreased ventricular function allows modification of the chemotherapy regimen, either by increasing the interval between doses or reducing the total cumulative dose of potentially toxic agents. Patients with left ventricular dysfunction or presenting with heart failure should be treated according to guidelines for heart failure.

In India, there are very few studies reporting acute cardiac dysfunction in patients receiving chemotherapy for breast cancer. The present study is planned with the aim of evaluating the incidence, clinical correlates, and response to therapy of cardiotoxicity in the Indian population.

## Materials and methods

This prospective study was conducted at Dayanand Medical College and Hospital (DMCH) in Ludhiana, Punjab, with the objective of detecting cancer therapy-related cardiac dysfunction in patients with breast cancer treated between March 1, 2020, and October 31, 2021, in the outpatient department (OPD) and inpatient department (IPD).

All newly diagnosed breast cancer patients aged over 18 who visited the Oncology Wing of DMCH for chemotherapy during the specified period were enrolled, and follow-up was conducted. A total of 108 patients were initially enrolled, out of which seven were excluded and four were lost to follow-up. Therefore, data from 97 patients was analyzed.

The inclusion criteria for this study encompassed patients with histologically proven breast cancer of any stage and aged over 18 years. Exclusion criteria were defined as follows: patients below 18 years of age, pregnant and lactating women, individuals with a past medical history of valvular heart disease, cardiomyopathy, congestive heart failure (CHF), ischemic heart disease, symptomatic arrhythmia, and pericardial diseases. Additionally, patients with advanced metastatic disease who were critically ill were excluded from the study. These criteria were established to ensure the homogeneity of the study population and to mitigate potential confounding factors.

This study received approval from the institute's ethical committee, and all patients were enrolled after obtaining informed consent. Following the acquisition of written informed consent, patients underwent a detailed history-taking process, with particular emphasis on risk factors for ischemic heart disease (IHD) such as diabetes mellitus, hypertension, obesity, dyslipidemia, and a family history of premature coronary artery disease (CAD). A comprehensive history and examinations of the cardiovascular system were conducted at baseline and during each follow-up visit. A thorough general physical examination, including assessments of blood pressure and pulse rate, was performed. Cardiovascular examinations were conducted to identify heart sounds, murmurs, and pericardial rubs.

Baseline investigations, including a complete blood count, renal function test, liver function test, chest X-ray, 12-lead electrocardiography (ECG), and 2D echocardiography, were conducted. Chemotherapy, administered in four to six cycles as determined by treating oncologists, was closely monitored. The timing of the first anthracycline dose and cumulative dose (mg/m2) were calculated to equivalent doxorubicin dose.

Cardiotoxicity was defined as a decrease in left ventricular ejection fraction (LVEF) by more than 10 percentage points from the baseline or less than 50%, or a relative decrease from baseline in global longitudinal strain (GLS) by more than 15% [13]. Echocardiography was performed before starting chemotherapy, midway through, and at the end of chemotherapy. Patients who developed left ventricular (LV) dysfunction underwent a fourth echocardiography three months after chemotherapy. Affected patients were managed according to heart failure guidelines. The same physician performed echocardiography for each patient at different time points. Various echocardiographic parameters, including M Mode, 2D echocardiography, Doppler echocardiography, tissue Doppler, global longitudinal strain (GLS), and global circumferential strain (GCS), were measured as shown in Table 1.

**Table 1:** Normal values for females LV: left ventricle; BSA: body surface area; EDV: end-diastolic volume; ESV: end-systolic volume; LVEF: left ventricular ejection fraction; LA: left atrium [14,15]

Parameters	Normal Range for Females
LV internal dimension	
Diastolic dimension (mm)	37.8-52.2
Systolic dimension(mm)	21.6-34.8
LV volume	
LV EDV (ml)	46-106
LVESV (ml)	14-42
LV volumes normalized to BSA	
LV EDV (ml/m^2^)	29-61
LV ESV (ml/m^2^)	8-24
LVEF (biplane, %))	54-74
LA volume/BS (ml/m^2^)	16-34
2D method	
LV mass(g)	66-150
LV mass/BSA(g/m^2^)	44-88
Septal thickness (cm)	0.6-0.9
Posterior wall thickness (cm)	0.6-0.9

LV volumes were measured using the modified Simpson's biplane method of discs, and LVEF was calculated accordingly. Additionally, diastolic function was assessed using trans-mitral flow parameters, and the left atrium (LA) maximum volume was measured using the biplane area-length method. A 2D strain analysis was utilized to measure the global longitudinal systolic strain and the global circumferential strain.

Statistical analysis was conducted using IBM SPSS Statistics for Windows, Version 21 (Released 2012; IBM Corp., Armonk, New York, United States). Data were described in terms of range, mean ± standard deviation, median, frequencies, and relative frequencies as appropriate. The comparison of quantitative variables was performed using the paired t-test, with a probability value (p-value) less than 0.05 considered statistically significant.

## Results

This study was conducted at a tertiary care hospital and involved 108 female patients with a histologically proven diagnosis of breast cancer. Seven patients were initially excluded, and an additional four were lost to follow-up. Among the remaining 97 patients, baseline characteristics were assessed, and 2D echocardiography was performed. Follow-up evaluations were conducted throughout the study duration.

All patients underwent three echocardiography sessions: the first before initiating chemotherapy, the second midway through the chemotherapy regimen, and the third upon completion of chemotherapy. Patients who developed left ventricular dysfunction received heart failure treatment and underwent a fourth echocardiography after a three-month follow-up period.

Distribution of breast cancer patients

Out of the total 97 female patients with breast cancer, the majority fell into the age group of less than 50 years (48.5%). In the 51-60 years age group, there were 32 patients (33%), while 18 patients (18.6%) were in the over-60 age group. Among the 97 patients, six (6.2%) were estrogen receptor (ER) positive, and the remaining 91 (93.8%) were ER-negative. Out of the total patients, 14 (14.4%) were progesterone receptor (PR) positive, while 83 (85.6%) were PR-negative. Regarding human epidermal growth factor receptor 2 (HER2) distribution, 37 (38.1%) patients were HER2 positive, and the remaining 60 (61.9%) were HER2 negative.

Cancer stage distribution among the patients was as follows: 42 (43.3%) had stage III cancer, 37 (38.1%) had stage II cancer, nine (9.3%) had stage I cancer, and six (6.2%) had stage IV cancer. Additionally, three (3.1%) patients had stage X cancer.

In terms of tumor, node, metastasis (TNM) staging, 55 (56.7%) patients had T2 stage, 28 (28.9%) had T3 stage, nine (9.3%) had T4 stage, and five (5.2%) had T1 stage. Regarding nodal staging, 36 (37.1%) had N1 stage, 24 (24.7%) had N0 stage, 22 (22.7%) had N2 stage, and 15 (15.5%) had N3 stage. Only four (4.1%) patients had M1 stage, while 93 (95.9%) had M0 stage.

All 97 (100%) patients underwent surgery before starting chemotherapy. Of these patients, 54 (55.6%) received radiotherapy, while 43 (44.3%) did not receive radiotherapy.

Left ventricular dysfunction distribution in breast cancer patients

Out of the total 97 patients with breast cancer, 13 (13.4%) developed LV dysfunction, while 84 (86.6%) remained normal.

Age group, hypertension, and surgical extent analysis

In the present study, there was no significant difference between various age groups (P=0.89). Among the 13 patients who developed LV dysfunction, six (46.2%) were in the age group of less than 50 years, five (38.5%) were from the 51-60 year age group, and two (15.4%) were >60 years of age. In the non LVD group, 41 (48.8%) patients were <50 years old, 27 (32.1%) were in the 51-60 years age group, and 16 (19%) were >60 years old, as shown in Table 2.

**Table 2:** Analysis of age group, hypertension, and surgical extent in LV dysfunction distribution LVD: left ventricular dysfunction; LV: left ventricle

Parameters		Non LVD (n=84)		LVD (n=13)			Total	Chi-square value		p-value
Age group (years)	<50	41	48.8%	6	46.2%		47	0.234		0.89
	51-60	27	32.1%	5	38.5%		32			
	>60	16	19.0%	2	15.4%		18			
Hypertension	No	84	100.0%	11	84.6%		95		13.195	0.017
	Yes	0	0.0%	2	15.4%		2			
Surgical extent wise	Left	52	61.9%	11		84.6%	61		14.676	0.001
	Right	32	38.1%	2		15.4%	34			

Regarding hypertension distribution, a significant difference was observed between the two groups (P=0.017). Out of 13 patients who developed LVD, only two (15.4%) were hypertensive, while 11 (15.4%) were non-hypertensive. In the non-LVD group, all 84 (100%) patients were non-hypertensive. No patient was found to be diabetic in either group, as shown in Table 2.

Similarly, a significant difference was noted between the two groups in terms of surgical extent distribution (P=0.001). Out of 13 patients who developed LVD, 11 (84.6%) had left breast involvement, while two (15.4%) had right breast involvement. Among the other 84 patients, 52 (61.9%) had left breast involvement, while 32 (38.1%) had right breast involvement, as shown in Table 2.

Receptor distribution analysis in left ventricular dysfunction among breast cancer patients

In our study, no significant differences were found between the two groups regarding the distribution of estrogen receptors (ER) (P=0.030). Among the 13 LV dysfunction patients, 10 (76.9%) were ER-negative, while three (23.1%) were ER-positive. In the LV dysfunction group, 81 (96.4%) were ER-negative, and three (3.6%) were ER-positive. Similarly, no significant differences were observed in the progesterone receptor (PR) distribution (P=0.341). Among the 13 LV dysfunction patients, 10 (76.9%) were PR-negative, and three (23.1%) were PR-positive. Among the rest, 73 (86.9%) were PR-negative, and 11 (13.1%) were PR-positive. Additionally, there were no significant differences in the human epidermal growth factor receptor 2 (HER2) distribution (P=0.551). In the LV dysfunction group, seven (53.8%) were HER2-negative, and six (46.2%) were HER2-positive. Among the others, 53 (63.1%) were HER2-negative, and 31 (36.9%) were HER2-positive, as shown in Table 3.

**Table 3:** Receptor distribution analysis in left ventricular dysfunction (LVD) among breast cancer patients

Parameter	Status	Non LVD (n=84)		LVD (n=13)		Total	Chi-square value	p-value
ER+	Negative	81	96.4%	10	76.9%	91	7.381	0.030
	Positive	3	3.6%	3	23.1%	6		
PR+	Negative	73	86.9%	10	76.9%	83	0.908	0.341
	Positive	11	13.1%	3	23.1%	14		
HER2+	Negative	53	63.1%	7	53.8%	60	0.408	0.551
	Positive	31	36.9%	6	46.2%	37		

Distribution of cancer stage and TNM staging in left ventricular dysfunction among breast cancer patients

In the study, no significant differences were found between the two groups regarding cancer stage distribution (P=0.341) or TNM staging distribution (P=0.066 and P=0.470, respectively). Among the 13 patients with LV dysfunction, stages III and II were predominant in cancer stage distribution, while T2 was the most common in TNM staging. However, no distinct patterns emerged in either group, suggesting a similar distribution across the LV dysfunction and non-LV dysfunction groups. Furthermore, in TNM staging, no significant differences were observed in N staging (P=0.470) or M staging (P=0.487), indicating a consistent distribution across both groups as shown in Table 4.

**Table 4:** Distribution of cancer stage and TNM staging in LV dysfunction among breast cancer patients LV: left ventricle; TNM: tumor, node, metastasis

Staging		Non LVD (n=84)			LVD (n=13)		Total	Chi-square	p-value
										Cancer stage									
TNM staging	I	9	10.70%		0	0.00%	9	4.511	0.341
	II	33	39.30%		4	30.80%	37		
	III	35	41.70%		7	53.80%	42		
	IV	4	4.80%		2	15.40%	6		
	X	3	3.60%		0	0.00%	3		
T	1	3	3.60%		2	15.40%	5	7.183	0.066
	2	50	59.50%		5	38.50%	55		
	3	25	29.80%		3	23.10%	28		
	4	6	7.10%		3	23.10%	9		
N	0	23	27.40%		1	7.70%	24	2.527	0.47
	1	30	35.70%		6	46.20%	36		
	2	18	21.40%		4	30.80%	22		
	3	13	15.50%		2	15.40%	15		
M	0	81		96.40%	12	92.30%	93	0.484	0.487
	1	3		3.60%	1	7.70%	4		

Radiotherapy and chemotherapy distribution among left ventricular dysfunction patients

Out of the total of 97 patients, 54 received radiotherapy. Among the 13 patients with LV dysfunction, seven (53.8%) received radiotherapy, while among the 84 patients without LV dysfunction, 47 (56%) received radiotherapy. Regarding chemotherapy, one drug was received by six (6.1%) patients, two drugs by 58 (59.7%) patients, three drugs by 21 (21.6%) patients, and a four-drug combination by 12 (12.37%) patients. Doxorubicin was prescribed to 62 patients, with nine (69.2%) LV dysfunction patients and 53 (63.1%) patients remaining normal. Cyclophosphamide was prescribed to 64 patients, with 10 (76.9%) LV dysfunction patients and 54 (64.3%) patients remaining normal. Trastuzumab was given to 36 patients, with five (38.5%) LV dysfunction patients and 31 (36.9%) patients remaining normal. Carboplatin was given to 32 patients, with five (38.5%) LV dysfunction patients and 27 (32.1%) patients remaining normal. Paclitaxel was prescribed to 33 patients, with six (46.2%) LV dysfunction and 27 (32.1%) remaining normal. Docetaxel was given exclusively to the LV dysfunction group, with four out of 13 (30.8%) LV dysfunction patients receiving docetaxel, as shown in Table 5.

**Table 5:** Distribution of radiotherapy and chemotherapy drugs among patients who developed LV dysfunction LV: left ventricle

Therapy	Non LVD (n=84)		LVD (n=13)		Total
RT dosage Gy	47	56.0%	7	53.8%	54
Doxorubicin	53	63.1%	9	69.2%	62
Cyclophosphamide	54	64.3%	10	76.9%	64
Trastuzumab	31	36.9%	5	38.5%	36
Carboplatin	27	32.1%	5	38.5%	32
Paclitaxel	27	32.1%	6	46.2%	33
Docetaxel	0	0.0%	4	30.8%	4

Recovery from left ventricular dysfunction

Out of the total 13 patients who developed LV dysfunction, 10 patients (76.9%) successfully recovered, while three patients (23.1%) remained in LV dysfunction. This indicates a significant proportion of patients experiencing recovery from LV dysfunction. The data highlights the potential for improvement in LV function among patients undergoing treatment.

Serial echocardiography findings in patients with left ventricular dysfunction

**Figure 1:**
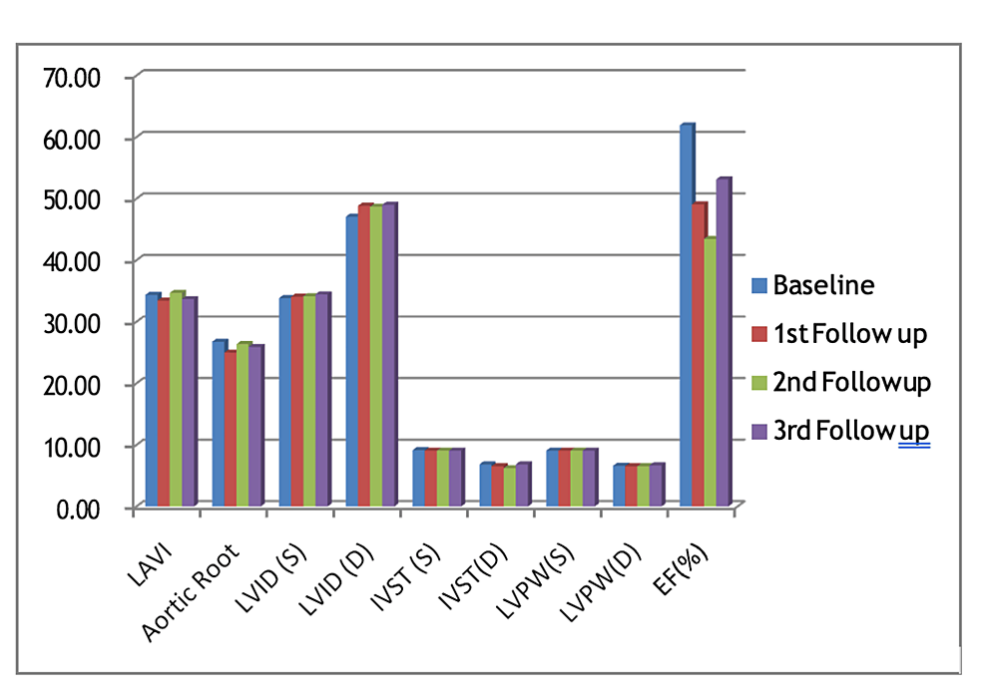
Serial echocardiography of patients who developed LV dysfunction LAVI: left atrial volume index; LVID(S): left ventricular internal diameter in systole; LVID(D): left ventricular internal diameter in diastole; IVST(S): interventricular septum thickness in systole; IVST(D): interventricular septum thickness in diastole; LVPW(S): left ventricular posterior wall thickness in systole; LVPW(D): left ventricular posterior wall thickness in diastole; EF(%): ejection fraction

The mean ejection fraction before initiating chemotherapy was 61.9%. The echocardiography conducted midway through chemotherapy revealed a mean ejection fraction of 49%, while at the end of chemotherapy, it decreased further to 43.4%. Subsequently, the patient who developed left ventricular (LV) dysfunction underwent a fourth echocardiography after three months, which showed an improved mean ejection fraction of 53.15%, as shown in Table 6 and Figure 1.

**Table 6:** Serial echocardiography of patients who developed LV dysfunction LAVI: left atrial volume index; LVID(S): left ventricular internal diameter in systole; LVID(D): left ventricular internal diameter in diastole; IVST(S): interventricular septum thickness in systole; IVST(D): interventricular septum thickness in diastole; LVPW(S): left ventricular posterior wall thickness in systole; LVPW(D): left ventricular posterior wall thickness in diastole; EF(%): ejection fraction; GLS(%): global longitudinal strain; GCS(%): global circumferential strain; MAPSE: mitral annular plane systolic excursion; DT(ms): deceleration time; e’(cm/s): early diastolic velocity; s’(cm/s): systolic velocity; E/e' ratio; PASP: pulmonary arterial systolic pressure

LVD (n=13)	First echocardiography		Second echocardiography		Third echocardiography		Fourth echocardiography	
	Mean	SD	Mean	SD	Mean	SD	Mean	SD
LAVI	34.38	0.77	33.46	0.78	34.69	1.75	33.69	1.97
Aortic root	26.77	1.42	25.00	1.63	26.38	0.77	25.92	0.86
LVID (S)	33.85	0.55	34.08	0.95	34.15	0.90	34.46	0.97
LVID (D)	47.08	1.12	48.85	1.77	48.69	1.65	49.00	1.08
IVST (S)	9.15	0.38	9.08	0.28	9.08	0.28	9.08	0.28
IVST(D)	6.85	1.07	6.54	0.88	6.23	0.83	6.85	1.14
LVPW(S)	9.08	0.28	9.08	0.28	9.08	0.28	9.08	0.28
LVPW(D)	6.62	0.87	6.54	0.88	6.54	0.88	6.69	0.85
EF(%)	61.92	0.64	49.08	12.97	43.46	4.05	53.15	6.39
GLS(%)	-19.18	1.20	-15.83	3.51	-14.46	1.52	-17.57	2.45
GCS (%)	-24.12	1.65	-20.58	2.80	-19.92	1.45	-22.12	2.09
MAPSE	12.92	1.12	10.38	2.02	9.31	0.75	10.85	1.41
DT(ms)	192.46	5.61	225.38	18.98	229.69	11.74	209.38	11.06
e'(cm/s)	10.23	0.60	8.62	1.94	8.77	1.01	9.77	1.30
s'(cm/s)	11.85	0.55	10.15	2.27	8.88	0.70	11.77	0.93
E/e'	4.23	0.60	11.92	2.10	10.08	2.25	7.85	2.08
PASP	0.00	0.00	3.69	9.16	6.23	10.79	2.54	6.23

**Figure 2:**
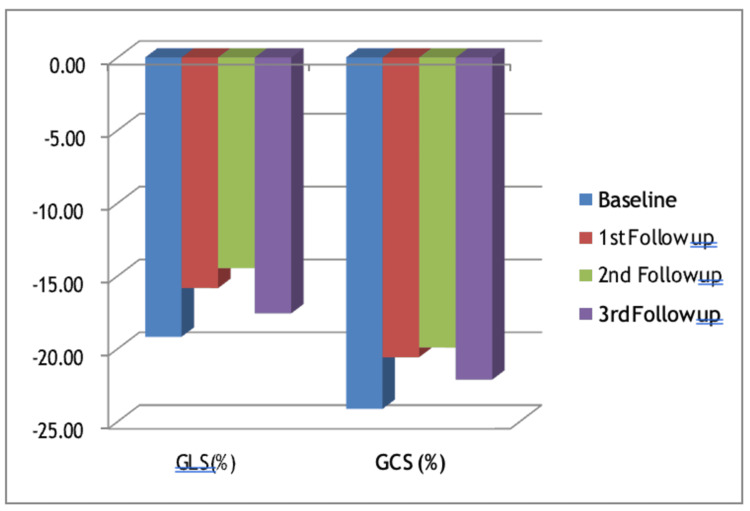
Changes in GLS and GCS across four sequential echocardiography examinations in LV dysfunction GLS: global longitudinal strain; GCS: global circumferential strain; LV: left ventricular

On the other hand, the global longitudinal strain (GLS) and global circumferential strain (GCS) during the first echocardiography were -19.18 and -24.12, respectively. During the second echocardiography, they improved to -15.83 and -20.58, while during the third echocardiography, they further ameliorated to -14.46 and -19.92, respectively. During the fourth echocardiography, the values were -17.57 for GLS and -22.12 for GCS, as shown in Figure 2.

Progression and severity of left ventricular dysfunction

**Figure 3:**
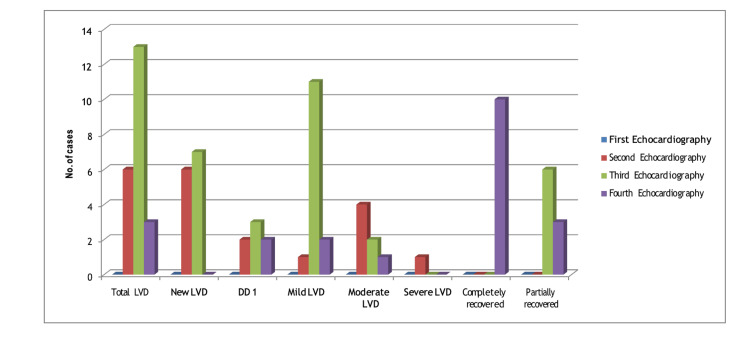
Progression and severity of LV dysfunction in subsequent echocardiography examinations DD: diastolic dysfunction; LVD: left ventricular dysfunction

The initial echocardiography revealed no evidence of LV dysfunction. However, during the second echocardiography, a total of six cases of LV dysfunction were identified, which increased to 13 in the third echocardiography. Out of these, three patients recovered, while 10 continued to exhibit LV dysfunction. Among the 13 cases, 11 had mild LV dysfunction, two had moderate LV dysfunction, and none showed severe LV dysfunction during the third echocardiography. Additionally, diastolic dysfunction grade 1 was observed in three patients during the same examination, as shown in Figure 3.

## Discussion

Cardio-oncology, an evolving discipline within cardiology, concentrates on detecting, monitoring, and treating chemotherapy and radiotherapy-induced cardiac dysfunction, particularly left ventricular (LV) dysfunction. Despite improved cancer survival rates, treatment-related side effects have amplified morbidity and mortality, with cardiovascular diseases emerging as major concerns. This study specifically examines chemotherapy-related acute cardiac toxicity in breast cancer patients in India, representing the first investigation of its kind in this population. While some literature predicts the incidence of acute cardiac toxicity, most studies focus on long-term follow-up, spanning over a year and encompassing both early and late chemotherapy toxicity.

A total of 108 newly diagnosed breast cancer patients initially presented at the oncology wing of a tertiary care hospital. Seven patients were excluded due to advanced metastatic disease with multi-organ failure, a baseline LV ejection fraction less than 55% on echocardiography, and prior chemotherapy. Consequently, 97 patients were enrolled. Four patients were lost to follow-up after initial radiotherapy. LV function was evaluated using echocardiography and global longitudinal strain before starting chemotherapy, with serial follow-up during mid- and end-of-chemo.

All patients underwent surgery, followed by an assessment by oncologists and radiotherapy specialists for radiotherapy necessity. Eligible patients received radiotherapy before chemotherapy, with 55.7% undergoing the radiotherapy. Baseline echocardiography was performed after radiotherapy, preceding chemotherapy initiation. Chemotherapy, typically four to six cycles, was determined by treating oncologists. Follow-up echocardiography was conducted during the mid- and end-of-chemotherapy cycles.

The risk assessment included detailed cardiovascular history, diabetes mellitus, hypertension, cancer staging, TNM classification, and baseline echocardiography. However, ECG, lipid profile, and cardiac biomarkers like brain natriuretic peptide (BNP) and troponin were not universally performed. Previous studies by Hyun JY et al. and Anita Boyd et al. did not include cardiac biomarkers and ECG [16,17]. While cardiac biomarkers may aid in the early detection of cardiac injury, evidence for chemotherapy interruption based on abnormal biomarkers remains inconclusive. Troponin I elevation, particularly in patients treated with trastuzumab after anthracycline exposure, indicates an increased risk of cardiac dysfunction. In the Cardinal et al. study, troponin I positivity was correlated with reduced LVEF persisting after chemotherapy cycles [7].

Chemotherapy drugs included doxorubicin, cyclophosphamide, trastuzumab, carboplatin, and paclitaxel. The distribution of chemotherapy drug combinations varied: 6.1% received one drug, 59.7% received two drugs, 21.6% received three drugs, and 12.4% received four drugs. Doxorubicin, cyclophosphamide, trastuzumab, carboplatin, and paclitaxel were received by 63.9%, 65.6%, 37%, 32.9%, and 34% of patients, respectively.

In our study, cardiotoxicity was defined as a decrease in LVEF >10 percentage points from baseline, or <50%, while subclinical LVD was defined as a relative decrease from baseline in global longitudinal strain (GLS) >15%. The incidence of acute cardiotoxicity was 13.4% in our study. Comparatively, Hyun JY et al. reported an incidence of LVD of 11.5%, while Anita Boyd et al. found that LVEF remained within the normal range in baseline and post-treatment evaluations [16,17].

In our study, 46.2% of patients developed LV systolic dysfunction during mid-chemotherapy, increasing to 53.8% at the end of chemotherapy. Diastolic dysfunction grade 1 was observed in 23% of patients. Similar findings were reported by Hyun JY et al. and Anita Boyd et al. Additionally, abnormal diastolic function was associated with a subsequent decrease in LVEF and worsening longitudinal strain over time, as reported by Jenica et al. [18].

Guidelines published by the American Society of Clinical Oncology (ASCO), the European Society for Medical Oncology (ESMO), and the European Society of Cardiology (ESC) provide detailed information on cardiovascular toxicities and offer practical approaches to diagnosis and management for physicians caring for cancer patients [19]. Patients at high risk of cardiac toxicity include those receiving high doses of anthracyclines, female gender, age over 65 or under 18 years, high doses of radiotherapy, concomitant treatment with trastuzumab, and pre-existing cardiac diseases, among others.

In the present study, the mean age in the LVD group was 50.44 and 50.77 in the non-LVD group, respectively. In the Hyun JY et al. study, the mean age in the LVD group was 56.7, and in the non-LVD group, 55.6 [16]. In the present study, 15.4% were in the LVD group, and no patient was hypertensive in the non-LVD group. In the Hyun JY et al. study, 17.1% of the LVD group and 23.5% of the non-LVD group were hypertensive [16].

In our study, none of the participants had diabetes, were smokers, or suffered from chronic kidney disease. However, previous research by Hyun JY et al. reported diabetic rates of 8.5% in the LV dysfunction (LVD) group and 9.7% in the non-LVD group [16]. Anita Boyd et al.'s study revealed that 22% of patients were hypertensive, 6% were diabetic, 25% were smokers, 19% had dyslipidemia, and 32% had a family history of cardiovascular disease [17].

Radiotherapy has been linked to cardiac toxicity, with some studies reporting a relative risk of fatal cardiovascular events between 1 and 2.2 in breast cancer patients [20]. The combination of left breast radiotherapy and chemotherapy poses a higher risk of cardiotoxicity, with marked interstitial myocardial fibrosis commonly observed. Our study found that 55.7% of patients received radiotherapy, with higher mean doses in the LVD group.

Regarding the cancer stage, our findings showed that 54% of the LVD group and 42% of the non-LVD group were in stage III, while 15.4% of the LVD group and 5% of the non-LVD group were in stage IV. These results are consistent with Hyun JY et al.'s study, which also found no consistent association between advanced cancer stage and LV dysfunction [16].

In our study, patients with LV dysfunction were more likely to be ER-positive (23% vs. 3.6%), PR-positive (23% vs. 13%), and HER2-positive (46% vs. 37%) compared to the non-LVD group. Left breast involvement was more common in the LVD group (84.6%) compared to the non-LVD group (62%), aligning with previous studies.

In terms of chemotherapy, our findings revealed that patients in the LVD group received higher percentages of doxorubicin, cyclophosphamide, trastuzumab, and paclitaxel compared to the non-LVD group. Anthracycline cumulative doses were within recommended limits, with no patients reaching the threshold associated with increased cardiac risk.

The mean ejection fraction decreased significantly during chemotherapy in the LVD group, while no substantial change was observed in the non-LVD group. Similarly, global longitudinal strain (GLS) reductions were observed in the LVD group, indicating a linear relationship with ejection fraction decline. GLS reductions were associated with clinical LV dysfunction.

Regarding cardiac function parameters such as left ventricular end-diastolic volume (LVEDV) and left atrial volume index (LAVI), our results were consistent with previous studies, showing no significant changes over time [16,17].

Patients with LV dysfunction received prompt heart failure therapy, including guidelines-directed medical therapy such as medications targeting the renin-angiotensin system (ACE inhibitors or ARBs) and beta-blockers, sometimes supplemented with diuretics, with 77% experiencing complete recovery on follow-up echocardiography after three months of chemotherapy. Hyun JY et al.'s study reported a similar recovery trend after 12 months of follow-up [16]. Patients who experienced partial recovery frequently had left breast involvement and were administered specific chemotherapy agents, underscoring the significance of tailoring treatment approaches based on the New York Heart Association (NYHA) class. This involves optimizing guideline-directed medical therapy, including ACE inhibitors, ARBs, beta-blockers, and diuretics, to enhance outcomes. Overall, our study underscores the importance of monitoring and managing cardiac toxicity in breast cancer patients undergoing chemotherapy and radiotherapy, especially those with specific risk factors.

The limitations of our study include its single-center focus, which may restrict the generalizability of findings to other populations, and the relatively small sample size of 97 patients. We enrolled patients across both the early and advanced stages of breast cancer, with those in the advanced stages receiving more cycles of chemotherapy and radiotherapy, potentially influencing the incidence of chemotherapy-related cardiac dysfunction (CTRCD). The study's duration was short, with patient follow-up limited to the chemotherapy period. Coronary angiography was not performed in the LV dysfunction (LVD) group, precluding the exclusion of coronary artery disease (CAD). Additionally, routine biomarkers such as troponin and BNP, as well as laboratory investigations like lipid profiles and blood glucose, were not included. Furthermore, sick patients with advanced disease were not part of our study cohort.

## Conclusions

In conclusion, despite limitations, our study highlights the significant incidence of acute cardiac toxicity among breast cancer patients. Essential hypertension, left breast involvement, and high doses of radiotherapy emerged as independent predictors of chemotherapy-related cardiac dysfunction (CTRCD). Close monitoring for CTRCD is crucial, especially for left breast-involved, hypertensive patients and those undergoing high-dose radiotherapy. Early diagnosis and prompt initiation of heart failure therapy led to complete recovery in 77% of patients, with 23% experiencing partial recovery. Notably, LV dysfunction was independent of cumulative chemotherapy doses. Patients with ER-positive, PR-positive, and HER2-positive status, as well as those in cancer stages III and IV, are at higher risk of developing LV dysfunction. Further studies are warranted to refine risk stratification and optimize treatment strategies for CTRCD in breast cancer patients.
